# Efficacy of Cabozantinib in Metastatic MiT Family Translocation Renal Cell Carcinomas

**DOI:** 10.1093/oncolo/oyac158

**Published:** 2022-08-18

**Authors:** Jonathan Thouvenin, Omar Alhalabi, Maria Carlo, Lucia Carril-Ajuria, Laure Hirsch, Nieves Martinez-Chanza, Sylvie Négrier, Luca Campedel, Dylan Martini, Delphine Borchiellini, Jad Chahoud, Massimo Lodi, Philippe Barthélémy, Elshad Hasanov, Andrew W Hahn, Thierry Gil, Srinivas R Viswanathan, Ziad Bakouny, Pavlos Msaouel, Mehmet Asim Bilen, Toni K Choueiri, Laurence Albiges, Nizar M Tannir, Gabriel G Malouf

**Affiliations:** Institut de Cancérologie Strasbourg Europe (ICANS/HUS), Strasbourg, France; Institut de Cancérologie des Hospices Civils de Lyon, Lyon, France; Dana-Farber Cancer Institute (DFCI), Boston, MA, USA; MD Anderson Cancer Center (MDACC), Houston, TX, USA; Memorial Sloan Kettering Cancer Center, New York, NY, USA; Institut Gustave Roussy, Villejuif, France; Dana-Farber Cancer Institute (DFCI), Boston, MA, USA; Institut Jules Bordet, Bruxelles, Belgium; Université Claude Bernard, Centre Léon Bérard, Lyon, France; AP-HP, Groupe hospitalier Pitié-Salpêtrière, Paris, France; Winship Cancer Institute of Emory University, Atlanta, GA, USA; Antoine Lacassagne Cancer Center, Nice, France; Department of Genitourinary Oncology, Moffitt Cancer Center, Tampa, FL, USA; Institut de Cancérologie Strasbourg Europe (ICANS/HUS), Strasbourg, France; Institut de Cancérologie Strasbourg Europe (ICANS/HUS), Strasbourg, France; MD Anderson Cancer Center (MDACC), Houston, TX, USA; MD Anderson Cancer Center (MDACC), Houston, TX, USA; Institut Jules Bordet, Bruxelles, Belgium; Dana-Farber Cancer Institute (DFCI), Boston, MA, USA; Dana-Farber Cancer Institute (DFCI), Boston, MA, USA; MD Anderson Cancer Center (MDACC), Houston, TX, USA; Winship Cancer Institute of Emory University, Atlanta, GA, USA; Dana-Farber Cancer Institute (DFCI), Boston, MA, USA; Institut Gustave Roussy, Villejuif, France; MD Anderson Cancer Center (MDACC), Houston, TX, USA; Institut de Cancérologie Strasbourg Europe (ICANS/HUS), Strasbourg, France

**Keywords:** MiT family translocation renal cell carcinoma, cabozantinib, non-clear cell renal cell carcinoma

## Abstract

**Background:**

MiT family translocation renal cell carcinoma (TRCC) is a rare and aggressive subgroup of renal cell carcinoma harboring high expression of c-MET. While TRCC response rates to VEGF receptor tyrosine kinase inhibitors (TKIs) and immune checkpoint inhibitors are limited, efficacy of cabozantinib (a VEGFR, MET, and AXL inhibitor) in this subgroup is unclear.

**Methods:**

We performed a multicenter, retrospective, international cohort study of patients with TRCC treated with cabozantinib. The main objectives were to estimate response rate according to RECIST 1.1 and to analyze progression-free survival (PFS) and overall survival (OS).

**Results:**

Fifty-two patients with metastatic TRCC treated in the participating centers and evaluable for response were included. Median age at metastatic diagnosis was 40 years (IQR 28.5-53). Patients’ IMDC risk groups at diagnosis were favorable (9/52), intermediate (35/52), and poor (8/52). Eleven (21.2%) patients received cabozantinib as frontline therapy, 15 (28.8%) at second line, and 26 (50%) at third line and beyond. The proportion of patients who achieved an objective response was 17.3%, including 2 complete responses and 7 partial responses. For 26 (50%) patients, stable disease was the best response. With a median follow-up of 25.1 months (IQR 12.6-39), median PFS was 6.8 months (95%CI 4.6-16.3) and median OS was 18.3 months (95%CI 17.0-30.6). No difference of response was identified according to fusion transcript features.

**Conclusion:**

This real-world study provides evidence of the activity of cabozantinib in TRCC, with more durable responses than those observed historically with other VEGFR-TKIs or ICIs.

**Implications for Practice**

MiT family translocation renal cell carcinoma (TRCC) is an aggressive renal cell carcinoma subtype, harboring high expression of c-MET. Validated treatment options in the metastatic setting are lacking. The activity of cabozantinib, a MET targeted agent in the treatment of metastatic TRCC patients is unknown. In this multicentric international retrospective study including 52 adults metastatic TRCC patients, 17.3% patients achieved an objective response and 26 (50%) patients had stable disease as best response. Median progression-free survival was of 6.8 months. Cabozantinib demonstrates clinical activity in the treatment of advanced TRCC and could be preferentially used in this setting.

## Introduction

Microphtalmia transcription factor (MiT) family translocation renal cell carcinoma (TRCC) is a rare subtype of renal cell carcinoma (RCC) harboring chromosomal translocations in transcription factor E3 (*TFE3*) and transcription factor EB (*TFEB*) genes.^1,2^ TRCC comprises 1%-5% of RCCs and is usually seen in children, adolescents, and young adults.^3,4^ Considering its rarity, there are no approved therapies in the metastatic setting, and most of the data about antitumor efficacy arise from retrospective studies.^5^ Vascular endothelial growth factor receptor (VEGFR)–targeted therapies have been used, and first-line progression-free survival (PFS) from retrospective cohorts varied between 3 and 8.2 months, although the number of cases included in these studies was limited, ranging from 11 to 24 patients.^6-8^ In the largest cohort to date assessing the efficacy of VEGFR inhibitor as first-line treatment (*n* = 24), only 2 patients (10.5%) showed partial response and 15 patients (62.5%) exhibited disease progression as the best overall response.^6^ Likewise, response to immune checkpoint inhibitors (ICIs) was limited, with a median PFS of 2.5 months.^6^

The current standard-of-care therapies for metastatic clear cell RCC (ccRCC) are combinations of ICIs^9^ and/or VEGFR tyrosine kinase inhibitors (TKIs).^10-12^ In non-clear cell RCC, response of patients to ICI seems to be limited, and the results of several trials comparing VEGR inhibitors with/without ICIs are awaited.^13,14^ Recently, Pal et al reported that cabozantinib treatment significantly improved PFS compared with sunitinib in patients with metastatic papillary RCCs, suggesting the benefit of MET-based inhibition in this setting.^15^

Similar to papillary RCC,^16^ TRCC harbors high expression of c-MET by immunohistochemistry (IHC).^17^ Furthermore, TFE3 fusion proteins have been shown to bind to the MET promoter, inducing MET autophosphorylation and activation of downstream signaling in the presence of hepatocyte growth factor (HGF).^18^ In malignant TRCC cell lines, MET knockdown induces a decrease in proliferation and viability.^18^ Therefore, targeting MET might represent an interesting option in the clinical setting. However, there is a knowledge gap as to whether MET inhibition will benefit patients with TRCC.

Cabozantinib is a TKI targeting *VEGFR*, *MET*, *RET*, *KIT*, and *AXL*^19^ currently approved for the treatment of metastatic RCC based on the results of two prospective studies, which only included patients with clear cell histology.^20,21^ Retrospective studies have reported the efficacy of cabozantinib in cohorts of non-clear cell RCC patients, with objective response rates (ORR) of approximately 30% and median times to treatment failure (TTF) of 6.7 months (95%CI 5.5-8.6).^22-24^ However, these studies included different histologies, precluding specific assessment of the benefit of cabozantinib in TRCC. We thus conducted an international, multicenter study to investigate the efficacy of cabozantinib in patients with TRCC. Treatment efficacy was also analyzed according to fusion partners in a subset of patients.

## Patients and Methods

### Patients

We performed a multicenter, international, retrospective study in 11 centers (5 in France, 1 in Belgium, and 5 in the US) between December 2011 and December 2020 to analyze the outcomes of metastatic TRCC patients treated with cabozantinib at any line of treatment. De-identified data from patients were collected and shared with the coordinating institution (Institut de Cancérologie Strasbourg, Strasbourg, France). Eligible patients were adults and had histologically confirmed TRCC by a dedicated genitourinary pathologist at each participating institution. Chromosomal translocations involving *TFE3* and *TFEB* were confirmed by florescent in situ hybridization (FISH) when possible; cases with suggestive morphology and nuclear TFE3 overexpression by IHC were also included. Patients had to be treated with cabozantinib for metastatic disease at any treatment line. Each of the participating centers obtained regulatory approval through their institutional guidelines. The study was performed in accordance with the ethical principles of the Declaration of Helsinki.

### Methods

Data were obtained from retrospective chart review by investigators at each institution between December 2011 and December 2020. Demographic, surgical, pathological, and systemic therapy data were recorded with uniform database templates to ensure consistent data collection. Starting dose of cabozantinib, dose modifications, reason for discontinuation, and adverse events were collected. Radiographic response was assessed locally at the participating center according to RECIST (Response Evaluation Criteria in Solid Tumors) version 1.1 by a collaborating radiologist. Clinical and radiographic assessments were done according to the participating center’s standard of care. The presence of grade ≥ 3 toxicities related to cabozantinib was retrospectively collected according to the National Institutes of Health Common Terminology Criteria of Adverse Events (CTCAE) version 5.0 by investigators at each of the participating centers. Data on tumoral molecular alterations obtained by next-generation sequencing and fusion-transcripts obtained by MSK-IMPACT were collected when available.

### Outcomes

The principal objective was to evaluate efficacy of cabozantinib in metastatic TRCC patients in terms of ORR, PFS, and overall survival (OS). ORR was defined as the proportion of patients who experienced a complete (CR) or partial response (PR) as best radiologic response. Clinical benefit included CR or PR and stable disease lasting more than 6 months. OS was calculated from treatment initiation until death or last follow-up. PFS was calculated from treatment initiation to treatment discontinuation for progressive disease or death.

### Statistical Analysis

Study endpoints were ORR according to RECIST 1.1 criteria, PFS, and OS. Statistical analyses and graphical plotting were carried out using R version 4.0.3 (2020-10-10) and R packages *tidyverse*, *survival*, and *survminer*. Survival analysis was carried out with Kaplan-Meier curves and log-rank tests. Risk estimates (hazard ratio [HR] with 95%CI) were calculated using a Cox proportional hazards regression model. For discrete variables, we performed 2-sided Fisher’s exact tests. For continuous variables, we performed Wilcoxon-Mann-Whitney tests.^25^

## Results

### Patient Characteristics

Fifty-five TRCC patients treated with cabozantinib at any time were identified. Three patients were excluded because they were not evaluable for response, and 52 patients were included in the study. Demographic and clinical characteristics of these patients are summarized in **Table 1**. Most of them were female (60% [31/52]) and had performance status 0-1 (69.3% [36/52]). Median age at diagnosis of metastasis was 40 years (IQR 28.5-53).

**Table 1.** Patient characteristics.

Characteristic	*n* = 52
Median age (range), years	40 (19-78)
Sex	
Female	31 (60%)
Male	21 (40%)
ECOG performance status	
0-1	36 (69.3%)
≥2	4 (7.6%)
NA	12 (23.1%)
IMDC prognostic group	
Favorable	9 (17.3%)
Intermediate	35 (67.3%)
Poor	8 (15.4%)
Previous nephrectomy	37 (71%)
Stage at diagnosis	
I–IIII	27 (52%)
IV	25 (48%)
Cabozantinib line	
1	11 (21.2%)
2	15 (28.8%)
>2	26 (50%)
FISH	
Positive	40 (77%)
Not available	12 (23%)
Translocation	
*TFE3*	46 (88.5%)
*TFEB*	6 (11.5%)
Metastatic sites	
Retroperitoneal lymph nodes	27 (52%)
Mediastinal lymph nodes	17 (32.7%)
Lung	28 (53.8%)
Bone	22 (42.3%)
Liver	18 (34.6%)
Brain	4 (7.7%)
Nature of prior systemic therapies, (*n* = 41)	
VEGF TKI monotherapy	33 (80%)
Immune checkpoint inhibitor monotherapy	20 (49%)
Combination ICI and VEGF TKI	6 (14.6%)
Combination ICI	7 (17%)

Abbreviations: ECOG, Eastern Cooperative Oncology Group; IMDC, International Metastatic Renal Cell Carcinoma Database Consortium; FISH, florescent in situ hybridization; TKI, tyrosine kinase inhibitors; ICI, immune checkpoint inhibit; VEGF, vascular endothelial growth factor.

Overall, 6 patients (12.5%) had TFEB translocations and 46 (88.5%) had TFE3 translocations. For all patients, suggestive MiT family RCC morphology and nuclear TFE3 or TFEB immunohistochemistry were observed. The results of TRCC diagnosis by FISH were available and positive for 40 patients (77%), including TFE3 and TFEB. Among the TRCC cases with TFE3 fusions, fusion transcript identity was available for 10 patients. Four had ASPSCR1, 2 had PRCC, and one had FUBP1 fusion partners. For the 3 remaining patients, no fusion partners were detected, probably due to technical issues.

According to the International Metastatic Renal Cell Carcinoma Database Consortium (IMDC),^26^ 9 (17.3%), 35 (67.3%), and 8 (15.4%) patients had favorable-, intermediate-, and poor-risk disease, respectively. Median number of metastatic sites was 2 (range: 1-6). The most frequent metastatic sites were lymph nodes (63.5% [33/52]), lung (53.8% [28/52]), bone (42.3% [22/52]), and liver (34.6% [18/52]). Forty-one patients received cabozantinib as second (28.8% [15/52]) or subsequent line (50% [26/52]). Among them, 33 (80%) received prior VEGF TKI monotherapy, and 20 (49%) received prior ICI monotherapy.

### Clinical Outcomes

Median follow-up was 25.1 months (IQR 12.6-39). ORR was 17.3% (9/52 patients) including 2 CRs and 7 PRs (Table 2). Stable disease was the best response for 26 patients (50%), lasting more than 6 months for 15 patients (29%). Overall clinical benefit rate was 46% (24/52 patients). Seventeen patients (32.7%) experienced progression as best response. The 2 patients who experienced CR had TFE3 translocation confirmed by FISH. Both were classified as intermediate prognosis according to IMDC and relapsed more than 1 year after nephrectomy for localized disease. The first was treated in second line with cabozantinib, after experiencing stable disease lasting for 8 months under sunitinib; he finally progressed after 36 months under therapy. The second patient was treated in third line with cabozantinib after a partial response lasting one year under sunitinib as a first line, followed by a stable disease under nivolumab. After 37.7 months, the latter patient remained under therapy.

**Table 2.** Objective response to cabozantinib in the whole population (*n* = 52)

Outcomes	Objective response rate, *n* (%)
Best overall response	9 (17.3%)
Complete response	2 (3.8%)
Partial response	7 (13.5%)
Stable disease	26 (50%)
>6 months	15 (29%)
<6 months	11 (21%)
Progressive disease	17 (32.7%)
Clinical benefit	24 (46%)

**Figure 1.**
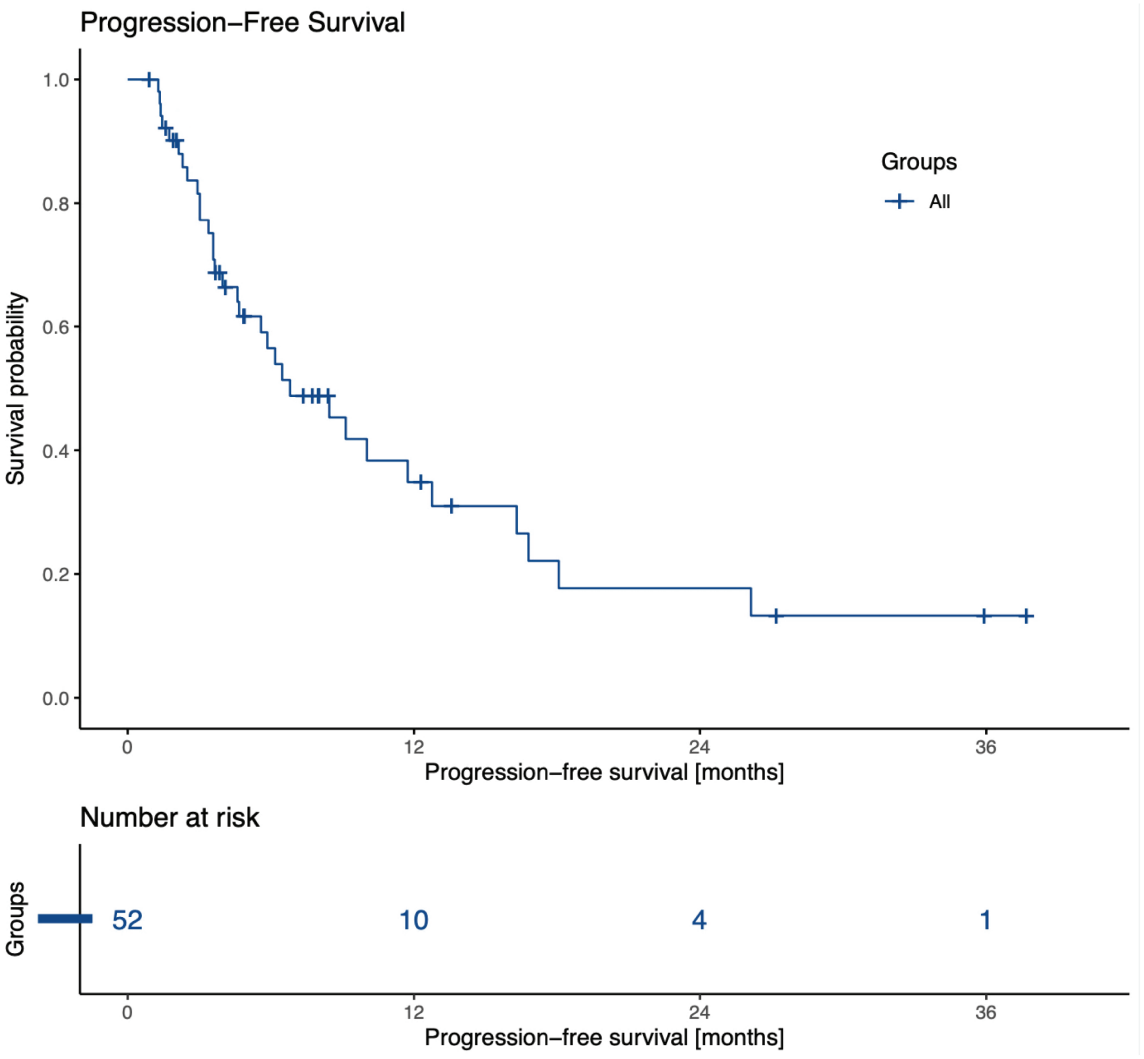
Progression-free survival in 52 advanced MiT family translocation RCCs treated with cabozantinib at any line.

**Figure 2.**
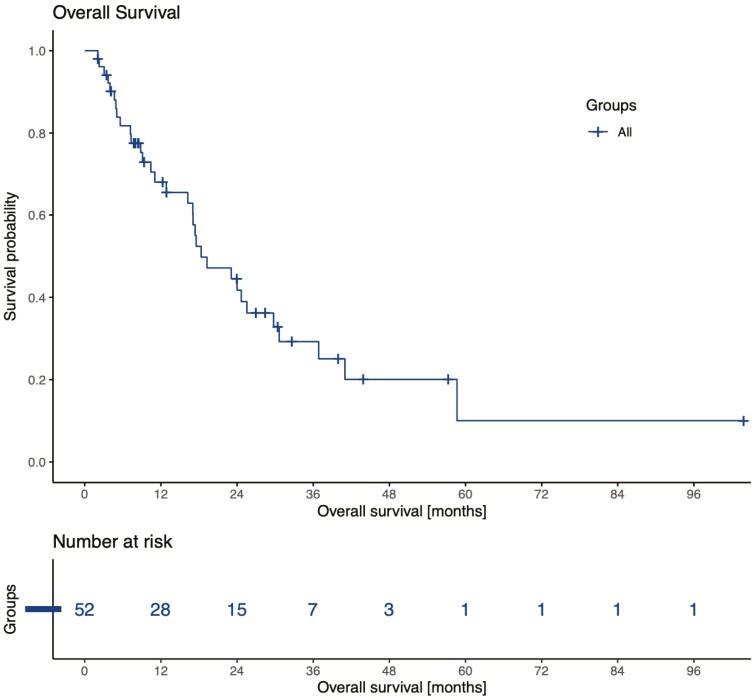
Overall survival in 52 advanced MiT family translocation RCCs treated with cabozantinib at any line.

At the time of analysis, 8 patients remained on therapy and 44 had discontinued cabozantinib, mainly for progression ([34/44] 77%) and toxicity ([9/44] 20.5%). Median PFS was 6.8 months (95%CI 4.6-16.3) (Fig. 1). Median OS was 18.3 months (95%CI 17.0-30.6) (Fig. 2), and 20 patients were still alive. According to IMDC classification, patients stratified as favorable risk (*n* = 9) had a median PFS of 6.2 months (95%CI 5.8-not reached) similar to that of intermediate- or poor-risk patients (*n* = 43), which was 6.8 months (95%CI 4.6-16.8). The initial dose of cabozantinib was 60 mg for the majority of patients ([36/52] 69%). Twenty patients (38.5%) needed a dose reduction, and 14 patients (27%) experienced grade 3 adverse events, most commonly palmar-plantar erythrodysthesia (6/14), fatigue (3/14), and diarrhea (3/14). One patient presented with heart failure and another presented with hepatitis related to cabozantinib. Nine (17%) of them had to stop treatment because of toxicities. There were no treatment-related deaths.

## Subgroups Analysis Regarding Efficacy of Cabozantinib

Exploratory subgroup analysis showed that patients with previous nephrectomy displayed a median PFS under cabozantinib of 6.5 months, which was longer than those without (4.7 months), HR 0.62 (95%CI 0.27-1.4) (*P = .*26) (Table 3). IMDC risk groups, line of treatment, and bone metastasis were not found to be associated with PFS. The 4 patients who had brain metastasis experienced a worse outcome than those without, with a median PFS of 3.8 (95%CI 2.1-not reached) versus 9.1 (95%CI 5.6-16.8), HR 3.1 (95%CI 1.1-9.3) (*P = .*029), respectively.

**Table 3.** Exploratory subgroups analysis of cabozantinib efficacy.

	Median progression-free survival, months (95%CI)	Hazard ratio, (95%CI)	*P*-value
IMDC (favorable vs intermediate/poor)	6.2 (5.8-not reached) vs 6.8 (4.6-16.8)	0.89 (0.33-2.4)	.82
Line of cabozantinib (1 vs ≥2)	11.7 (4.7-not reached) vs 6.5 (3.6-16.3)	0.59 (0.23-1.5)	.28
Prior nephrectomy (yes vs no)	6.5 (3.6-26.1) vs 4.7 (4.0–not reached)	0.62 (0.27-1.4)	.26
Bone metastasis (yes vs no)	6.8 (4-not reached) vs 6.5 (4.6-16.8)	0.78 (0.38–1.6)	.5
Brain metastasis (yes vs no)	3.8 (2.1-not reached) vs 9.1 (5.6-16.8)	3.1 (1.1-9.3)	.03

## Molecular Correlates of Response to Cabozantinib

*TFE3* fusion partner assessment was available for 10 patients using MSK-IMPACT. *ASPSCR1* was the most frequent partner identified (4/10), while *FUBP1* and *PRCC* were identified in 1 and 2 patients, respectively. For the 3 remaining patients, TFE3 fusion partner was not identified by MSK-IMPACT probably due to technical error. Regarding the fact that ASPSCR1 tumors were recently shown to harbor angiogenesis signature,^27^ we performed exploratory analysis to assess whether there are difference of response rate, PFS or OS in the 3 fusion transcript groups: *TFEB* (*n* = 6), other-*TFE3* (*n* = 4), and *ASPSCR1-TFE3* translocation RCC (*n* = 6). Median PFS was 6.8, 3.6, and 3 months in *TFEB*, other-*TFE3* translocation and *ASPSCR1-TFE3* translocation. Notably, no statistically significant difference was observed according to the fusion transcript.

## Discussion

Thanks to an international collaborative effort, this is, to the authors' knowledge, the largest retrospective study showing the activity of cabozantinib, an oral TKI targeting VEGFR, MET, and AXL, in TRCC. Cabozantinib showed encouraging antitumor efficacy regardless the line of treatment, with a median PFS of 6.8 months (95%CI 4.6-16.3) and 46% of patients experiencing clinical benefit. The efficacy observed was higher than historically reported results of VEGFR-TKI^6^ in a highly pretreated population, which varied between 3 and 8.2 months. Moreover, in our cohort, only 32.7% of patients presented progression as best response, when compared with the 62.5% with first-line VEGFR-TKI.^6^ No unexpected toxicities or treatment-related deaths were reported. This might be related to high expression of c-MET in these tumors.^17^ Furthermore, TFE3 fusion protein has been shown to bind to the MET promoter, inducing its auto-phosphorylation and activation of downstream signaling in the presence of hepatocyte growth factor (HGF).^18^ Thus, it is tempting to speculate that the activity of cabozantinib in this subgroup is driven by c-MET. Analysis of differentially expressed genes between TRCC and normal kidneys in Sun et al study confirmed overexpression of MET, in contrast to AXL and VEGFR.^27^ The activity of a selective MET inhibitor tivantinib has been tested in MiT associated tumors including only 6 TRCC patients. Among them 3 experienced stable disease as best response.^28^ Notably, our cohort included heavily pretreated patients, with 50% of patients receiving cabozantinib as a third line or more. In addition, the majority of our patients had intermediate- or poor-risk disease according to IMDC at diagnosis.

Cabozantinib has been approved in the treatment of advanced RCC according to the results of two randomized trials.^20,21^ METEOR,^21^ a phase III trial comparing cabozantinib with everolimus in pretreated patients, and CABOSUN,^20^ a phase II trial comparing cabozantinib with sunitinib in first line, both showed PFS and ORR improvements, but these studies only included clear cell histology. Recently, Martinez-Chanza et al^22^ reported activity of cabozantinib in 112 advanced non-clear cell RCC patients. A subgroup of 17 pretreated TRCC patients were included with an ORR of 29% and a median time to treatment failure of 8.3 months (95%CI 4.6-NA) in this subgroup. These data are consistent with ours and consolidate our findings, although the study did not report the criteria used for defining TRCC (ie, FISH) and no TFEB cases were included. Furthermore, according to the results of the PAPMET trial,^15^ cabozantinib is now approved in the first-line treatment of metastatic papillary RCC, a subtype of RCC driving by the MET signaling pathway.^29^

We also explored the efficacy of cabozantinib in TRCC with brain and bone metastasis. Indeed, the presence of brain metastasis is associated with significant morbidity and mortality in ccRCC.^30,31^ Cabozantinib was shown to be active in RCC brain metastasis with an observed intracranial response rate of 60%.^32^ However, in our cohort, the presence of brain metastasis was associated with a worse outcome, although only 4 patients were included for this analysis. The presence of bone metastasis is also associated with poor prognosis in RCC.^33^ Recent data have shown notable cabozantinib activity in this subgroup of patients.^34^ This might be explained by the key role of c-MET in modulating the activity of osteoblasts and osteoclasts. In our cohort, among the 22 patients presenting bone metastasis, the median PFS was 6.8 months (95%CI 4-NA), similar to those without.

TRCC is characterized by gene translocation involving *TFE3* and *TFEB* genes, with several partners evident in the data.^17^ Most frequently, *TFE3* fusion transcripts include *ASPSCR1*, *PRCC*, *SPPQ*, and *NONO*. However, whether there are distinct transcriptomic features associated with these partners remains unknown. Historically, *ASPSCR1* has been shown to be associated with the poorest outcome.^35^ Herein, among patients for whom transcriptomic data were available, almost the half of the patients harbored *ASPCR1* translocations, but no association with cabozantinib has been found.

Limitations of this study include potential selection bias due to the retrospective nature of the analysis. However, TRCC is rare, and the cohort included is representative of the daily clinical practice population. This study also lacked central pathological and radiographical review, which may have affected eligibility and tumor response assessment. However, patients were included in expert genitourinary oncology centers, which palliated these weaknesses.

## Conclusion

This multicenter, retrospective study, in the absence of available prospective data, provides evidence supporting the activity of cabozantinib in the control of advanced MiT family RCC. Given the rarity of this histological subtype of RCC, international collaboration and prospective studies are necessary to identify efficacious therapies for this rare disease that lacks prospectively validated treatment options.

## Data Availability

The data underlying this article cannot be shared publicly due to patient confidentiality. Gabriel G. Malouf and Jonathan Thouvenin have full access to all the data in the study and take responsibility for the integrity of the data and the accuracy of the data analysis.
